# Genome-Wide Analysis of Nucleotide-Level Variation in Commonly Used *Saccharomyces cerevisiae* Strains

**DOI:** 10.1371/journal.pone.0000322

**Published:** 2007-03-28

**Authors:** Joseph Schacherer, Douglas M. Ruderfer, David Gresham, Kara Dolinski, David Botstein, Leonid Kruglyak

**Affiliations:** 1 Lewis-Sigler Institute for Integrative Genomics, Princeton University, Princeton, New Jersey, United States of America; 2 Department of Ecology and Evolutionary Biology, Princeton University, Princeton, New Jersey, United States of America; 3 Department of Molecular Biology, Princeton University, Princeton, New Jersey, United States of America; Washington University in St. Louis School of Medicine, United States of America

## Abstract

Ten years have passed since the genome of *Saccharomyces cerevisiae*–more precisely, the S288c strain–was completely sequenced. However, experimental work in yeast is commonly performed using strains that are of unknown genetic relationship to S288c. Here, we characterized the nucleotide-level similarity between S288c and seven commonly used lab strains (A364A, W303, FL100, CEN.PK, ∑1278b, SK1 and BY4716) using 25mer oligonucleotide microarrays that provide complete and redundant coverage of the ∼12 Mb *Saccharomyces cerevisiae* genome. Using these data, we assessed the frequency and distribution of nucleotide variation in comparison to the sequenced reference genome. These data allow us to infer the relationships between experimentally important strains of yeast and provide insight for experimental designs that are sensitive to sequence variation. We propose a rational approach for near complete sequencing of strains related to the reference using these data and directed re-sequencing. These data and new visualization tools are accessible online in a new resource: the Yeast SNPs Browser (YSB; http://gbrowse.princeton.edu/cgi-bin/gbrowse/yeast_strains_snps) that is available to all researchers.

## Introduction

For decades, the budding yeast, *Saccharomyces cerevisiae*, has been used as a model organism for studying eukaryotic molecular and cell biology. Its primacy as a model system and its small and compact genome made it the best eukaryotic candidate for the early sequencing efforts. In 1996, the strain S288c, first isolated in the 1950's through genetic crosses by Robert Mortimer [1], became the first eukaryote to be completely sequenced [2]. The determination of this reference sequence facilitated the construction of the first whole genome microarrays [3] and the systematic deletions of all genes [4], as well as the first whole genome protein and genetic interaction maps [5], [6].

Despite the fact that most of the genomic information has been obtained from S288c, this strain is often not the ideal genetic background for studying particular aspects of biology. In fact, the S288c background has a number of drawbacks, such as low sporulation efficiency [7], an inability to grow on maltose [8] and failure to initiate filamentous growth upon nitrogen starvation [9]. For these reasons, other genetic backgrounds are used for physiological [10], genetic and genomic analyses [11]–[13] in many laboratories. These other genetic backgrounds are sometimes of known close (e.g. A363A and W303) or distant (SK1) genetic relatedness to S288c. However, for some experimentally important strains the relationship is unclear and largely undocumented.

The use of these different strains may contribute to the inconsistencies in some biological results because it is often assumed that the genomic sequence information of S288c can be extrapolated to other strains. In fact, even for strains closely related to S288c, the small number of sequence differences may still have important consequences for different biological pathways and phenotypes. Therefore, understanding genetic differences between strains has become extremely important. Mortimer and Johnson [1] traced a genealogy of the commonly used *S. cerevisiae* strains from knowledge of their history. More recently, Winzeler *et al.* [14] determined a subset of DNA sequence variation among a set of 14 *S. cerevisiae* strains using low-coverage oligonucleotide arrays and discovered 11,115 sites of variation among them. Although this study provided some insight into allelic differences between yeast strains, it was limited by the proportion of the genome covered by the array (∼16%) and the 25 bp resolution for localizing these variants.

We recently developed a method for characterizing nucleotide variation in the entire genome using 25mer oligonucleotide microarrays (Affymetrix yeast tiling arrays) that provide complete and redundant coverage of the ∼12 Mb *S. cerevisiae* genome [15]. This design provides for multiple measurements of each nucleotide's contribution to hybridization efficiency and therefore has the ability to detect the presence and location of single nucleotide polymorphisms (SNPs) and deletion events throughout the entire yeast genome with near nucleotide precision. We have employed this approach to characterize the nucleotide-level similarity and divergence between S288c and 7 commonly used lab strains (A364A, W303, FL100, CEN.PK, ∑1278b, SK1 and BY4716). The analyses revealed which genomic regions of each strain are derived from the reference strain and which regions are highly diverged, indicating a different ancestry. The data are presented in an online database, the Yeast SNPs Browser (YSB; http://gbrowse.princeton.edu/cgi-bin/gbrowse/yeast_strains_snps). YSB represents a valuable tool for the yeast community, enabling the development of genomic resources for other yeast strains and informing conventional molecular biology methods such as PCR primer and Southern blot probe design.

## Results and Discussion

### Frequency of SNPs between strains

For this study, 7 unsequenced laboratory strains, A364A, W303, FL100, CEN.PK, ∑1278b, SK1 and BY4716, were chosen because of their importance and frequent use in research (Table 1). For example, the SK1 background is used for studying sporulation and meiosis [7], [16], ∑1278b is used in pseudohyphal growth studies [17] and A364A is widely used in studies of the cell cycle [18]. As a control, we also included the strain BY4716 that is known to be nearly identical to S288c [19].

**Table 1:** Strains of *S. cerevisiae* used in this study

Strain	Number of SNPs*	Nucleotide divergence (%)**	Number of deletions***	Genotype	Reference
BY4716	39	0	1	Mat. α Lys2Δ0	[19]
A364A	4894	0.05	8	Mat. a ade1 ade2 ura1 his7 lys2 tyr1 gal1	[18]
W303	7955	0.08	7	Mat. a	[30]
FL100	15352	0.15	16	Mat. a	[31]
CEN.PK	13914	0.13	17	Mat. a	[10]
Σ1278b	25298	0.24	26	Mat. α lys2 ho::LYS2	[32]
SK1	37424	0.36	36	Mat. α	[33]

* number of calls at a prediction signal of 5.

** nucleotide divergence from S288C was estimated based on the number of calls at a prediction signal of 5 corrected for the 13.1% of the calls expected to be missed at this threshold (as estimated from the data for the fully sequenced strain YJM789)

*** number of deletions that are longer than 500 bp in size.

To characterize the genomes of these unsequenced laboratory strains at the nucleotide level, we hybridized genomic DNA from each strain to a high-density Affymetrix yeast tiling microarray and detected potential SNPs using the software package *SNPscanner* as described [15]. Where possible, we analyzed a prototrophic wildtype clone that was representative of the strain. The provenance of the strains is indicated in Table 1. *SNPscanner* detects SNPs based on up to 7 measurements of a nucleotide's effect on hybridization efficiency and calculates the log of the likelihood ratio (referred as the prediction signal) for the presence of a SNP at each nucleotide in the genome. The ability to detect a large number of SNPs in an entire diverged genome was tested in our previous study using the two completely sequenced strains, RM11_1a and YJM789. Analysis of a single hybridization of YJM789 with *SNPscanner* correctly detected 86.9% of 30,303 known SNPs at a prediction signal of 5, with only 7 false positives calls (i.e. a rate of 1 false positive/2 Mb) across the nonrepetitive portion of the genome.

We therefore extended this approach to obtain a nucleotide-resolution comparison of the commonly used laboratory strains with the S288c reference strain by analyzing data from a single hybridization of each strain. Using the same threshold settings (a prediction signal of 5 and heuristic filters) as reported previously [15], we found a moderately low number of predicted SNPs in the two strains closely related to S288c (4,894 calls in A364A and 7,955 calls in W303), whereas a higher number of SNPs was detected in the more distant strains (15,352 calls in FL100, 25,298 calls in ∑1278b and 37,424 calls in SK1) (Table 1). The divergence between the different strains and the reference varies between 0.05% to 0.36% of the genome. As expected, the number of detected SNPs for the BY4716 strain was very low (39 calls in total).

### Genome-wide distribution of SNPs

To identify strains that share a common recent ancestry with the reference strain, we examined the genome-wide distribution of variation for each strain. For strains derived from S288c, we expect a small number of SNPs nonrandomly distributed across the genome. By contrast, the strains unrelated to S288c should have a high number of SNPs distributed throughout the genome.

**Figure 1:**
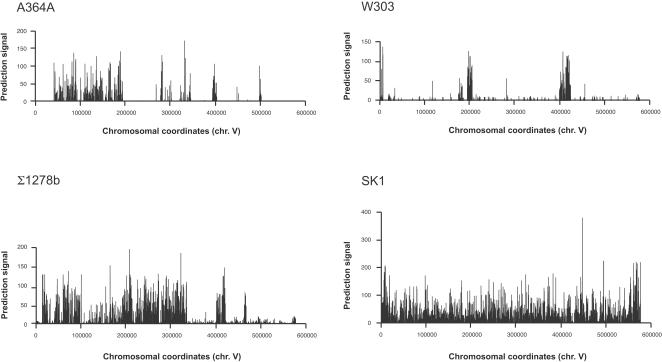
Prediction signal across chromosome 5 for the laboratory strains A364A, W303, Σ1278b and SK1. A364A and W303 genomes are mosaics of regions diverged from S288C, as indicated by extended regions of positive prediction signal for polymorphisms, interspersed with extended regions of sequence identity or low polymorphism, indicating common ancestry or recent divergence. The genomes of Σ1278b and SK1 show extensive divergence for most or all regions.

**Figure 2:**
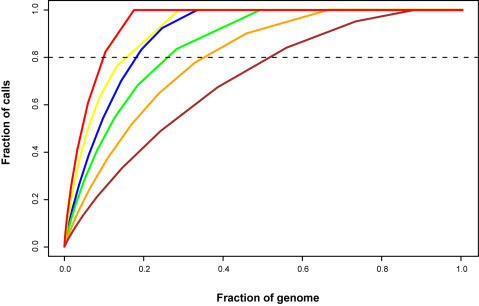
Genome-wide distribution of variation. Curves represent the fraction of the genome (X-axis) that contains a given fraction of all polymorphism calls (Y-axis) for the strains A364A, W303, CEN.PK, FL100, Σ1278b and SK1, respectively (from left to right).

The distribution of SNPs in A364A, W303 (both known to be the result of crosses between S288c and unrelated strains) and CEN.PK is indeed nonrandom. The genomes are mosaics of large regions identical to S288c interspersed with small regions of high sequence divergence (Fig. 1). In the strains closely related to S288c, variation was found in a small fraction of the genome, as previously described [14] (Fig. 2). For example, 80% of the SNPs in A364A, W303 and CEN.PK are found in 10%, 15% and 18% of the genome, respectively. By contrast, variation in SK1 is distributed relatively evenly throughout the genome.

**Figure 3:**
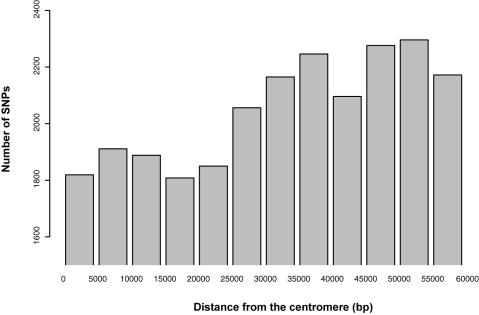
Distribution of variation by distance from centromere. The height of each bar represents the number of SNPs detected in the 3 strains that are not close relatives of S288c (FL100, ∑1278b and SK1) in a 5 kb bin, starting at the centromere and moving up to 55 kb away in either direction, pooled across all chromosomes (that is, the first bin contains all SNPs within 0–5 kb of a centromere, the next bin contains all SNPs 5–10 kb from the centromere, and so on out to the last bin which contains all SNPs 55–60 kb from the centromere). The polymorphism rate is lower in the 25–30 kb closest to the centromere.

For the strains that are clearly not close relatives of S288c, we asked if SNPs are unevenly distributed across the chromosomes. In all strains, variation is distributed across the genome, but decreases with proximity to the centromere (Fig. 3). The centromeric regions (within 20 kb of the centromeres) show a lower-than-average variability. This observation is possibly linked to the lack of DNA double-strand breaks (i.e the presence of meiotic recombination coldspots) near the centromeres [20]–[22]. Because of a reduced number of cross-overs and gene conversion events, these regions are less polymorphic, a result that is consistent with population genetic theory and observations in other organisms (e.g. Drosophila) [23].

By contrast, it is well known that subtelomeric regions that undergo frequent recombination events display increased variability at the sequence level [24]–[26]. However, these regions are rich in redundant sequences that are responsible for high levels of false positives using our strategy. In an effort to reduce false positives, *SNPscanner* removes all probes that are repeated on the array, thereby removing many of these regions from the initial analysis. With these regions removed, we fail to detect a higher proportion of genetic variability at the regions located near the telomere. When we then include these repeated regions, we see a marked increase in SNP calls near the telomere. However, we are currently unable to determine the percentage of these that are real versus false positives.

### Deletion variants in the surveyed strains

As shown in our previous study [15], hybridization of genomic DNA to the yeast tiling arrays can be used to detect deletions and predict the breakpoints with a resolution of a few basepairs. Therefore, these data provide a precise and global view of the deletion events in the laboratory strains examined. To distinguish regions that are deleted from those that are present but highly polymorphic-situations that result in similar *SNPscanner* profiles-we restricted our analysis to deleted regions that are longer than 500 bp in size (Table 1 and S1).

**Figure 4:**
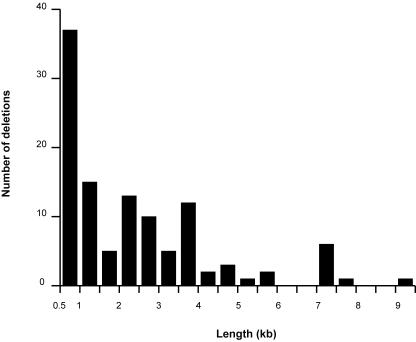
Size distribution of the deletion variants identified. To identify a confident set of the deletions, we limited analysis to those greater than 500 bp in size.

A total of 113 deletions met this criterion in a comparison between the reference strain and the other strains of this study. The number of deletions varied from 1 in the BY4716 strain to 36 in the SK1 strain. As expected, only the known engineered deletion of the *LYS2* gene was detected in the BY4716 strain. The mapped deletions range from 0.5 to 9.1 kb, with the majority falling between 0.5 and 1 kb (Fig. 4).

We identified all the genes that are involved in these deletion events. In total, 87 genes carry a whole or partial deletion in at least one strain (S1). Twenty-five genes have partial deletions, whereas 62 genes are deleted in full. We compared the function of the deleted genes on the basis of their Gene Ontology (GO) annotations [27] by using the GO Term Finder from the *Saccharomyces* genome database [28]. We found no significant enrichment with respect to biological function. A large proportion of the deleted genes (∼26%) have not yet been assigned a biological function. However, some of them play an important role in metabolism and are involved in transporter activity (*SEO1*, *ARR3*, *AGP3*, *MAL11*, *QCR8*, *HXT17*, *OPT2* and *SGE1*genes). All of these genes except for *QCR8* are located within 25 kb of the chromosome ends, consistent with a relationship between adaptation and subtelomeric regions, as previously reported [14].

### Sequencing strategy for the related S288c strains

Random shotgun whole genome sequencing is of unquestioned utility for de novo determination of an organism's genome. However, once a reference genome sequence has been determined for a species, applying this approach to re-sequence other closely related members of the population represents a redundant and inefficient approach. Although our method for comparing genomes at nucleotide resolution does not provide the actual DNA sequence of a genome, it does offer one approach to a more efficient resequencing of closely related strains. In this scheme, we suggest that initial determination of regions that are diverged from the reference genome using *SNPscanner* can then be followed by targeted re-sequencing of these regions.

We propose that this approach is applicable for strains A364A, W303 and CEN.PK, where the majority of variation was found in a small fraction (<20%) of the genome. By selecting and sequencing only the 1 kb regions of high diversity as identified by *SNPscanner*, we can obtain sequence information for 90% of a yeast strain's genome by sequencing only 1–3 megabases, substantially minimizing cost and effort (Table 2). Although these strains are experimentally important, it is unlikely that they will be of high priority for high coverage sequencing projects. Therefore, we propose that this approach will be informative until whole genome sequencing is greatly reduced in cost. This approach is limited to strains that are clear relatives of S288c, with much of their genomes being identical and the differences clustered into small regions. In addition, this approach will not identify regions of the genome (insertions) that are present in the sample strain but unrepresented in the reference strain.

**Table 2:** Sequencing strategy for the S288c related strains

Strain	% variation covered	Number of 1kb regions to be sequence	% of genome to be sequenced
A364A	90	1,618	13
W303	90	2,676	22
CEN.PK	90	2,795	23

### The Yeast SNPs Browser

**Figure 5:**
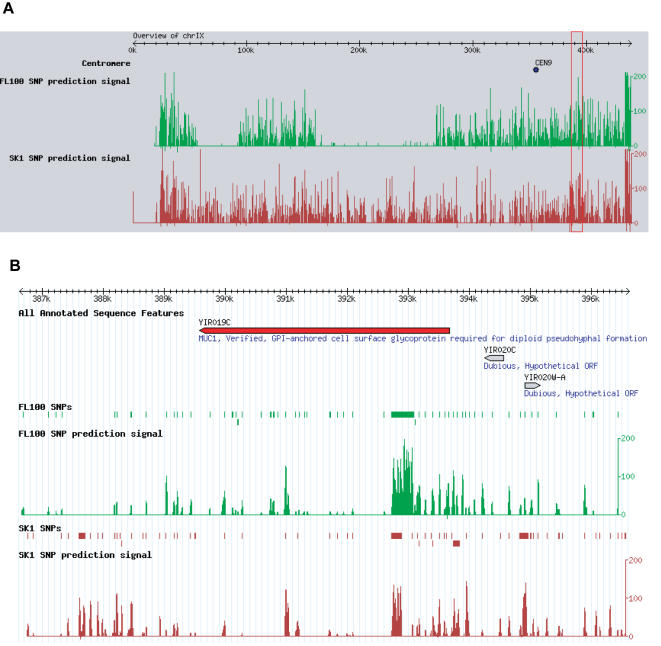
Yeast SNPs Browser screenshots. (A) Overview track of the entire chromosome IX for the FL100 and SK1 strains, showing the prediction signal across the chromosome. (B) Zoom-in of the region that includes the *MUC1* gene, showing the SNPs called and the associated prediction scores.

To allow researchers to easily visualize and explore these data we have created a database, the Yeast SNPs Browser, which can be used to view all the SNPs and deletions distinguishing these strains, along with the prediction signal across every base in the genome. We used the Generic Genome Browser (GBrowse), a web-based application for displaying chromosomal features [29], to provide user-friendly access to the data via the web. The Yeast SNPs Browser provides a way to view the putative SNPs in all the strains assayed in the context of the S288C genome. One can search by gene, feature name or oligonucleotide sequence, browse chromosomal regions, and zoom from an overview of an entire chromosome to the individual nucleotide level (Fig. 5). By zooming in to about 100 bp resolution, one can see the nucleotide sequence of the S288C reference strain in the region of the SNP. In addition, the prediction score is available as a graph across every base in the genome (Fig. 5).

### Conclusion

There are several *S. cerevisiae* strains that are commonly used in laboratory experiments, some of which are distantly related to the sequenced reference strain S288c. Understanding this variation is important for interpreting biological results from experiments performed with these strains. Using yeast tiling arrays, we have defined the genetic variation between the common laboratory strains of yeast at the nucleotide level in a reliable, cost-effective manner. We have also created a simple and visually pleasing resource for researchers. These data provide a valuable tool for the yeast community. They should inform experimental work that is reliant on knowledge of sequence such as the design of primers for PCR reactions and probes for Southern analysis. For studies of gene expression in the different laboratory strains, these data can also be useful as a source of information to design a “universal” microarray by selecting probes in regions of complete or high sequence similarity, in order to avoid any potential confounding effects on hybridization of strain-specific sequence variation. Finally, these data constitute the first step toward discovering the underlying genetic differences that contribute to the phenotypic differences among these strains.

## Materials and Methods

### Yeast strains

Strains used in this study are listed in Table 1.

### Sample preparation and hybridization on arrays

Yeast strains were grown in yeast extract, peptone, and dextrose (YPD) medium. Total genomic DNA was purified from 30 ml YPD culture using Qiagen Genomic-Tips 100/G and Genomic DNA Buffers as per the manufacturer's instructions. Genomic DNA was digested with DNaseI, labeled and hybrizided to Affymetrix Yeast Tiling Arrays as described in Gresham *et al.* [15].

## Supporting Information

Table S1 Genes carrying a whole or partial deletion in the surveyed strains
(0.02 MB DOC)
